# Abnormal Resting-State Functional Connectivity in the Whole Brain in Lifelong Premature Ejaculation Patients Based on Machine Learning Approach

**DOI:** 10.3389/fnins.2019.00448

**Published:** 2019-05-08

**Authors:** Ziliang Xu, Xuejuan Yang, Ming Gao, Lin Liu, Jinbo Sun, Peng Liu, Wei Qin

**Affiliations:** ^1^Engineering Research Center of Molecular and Neuro Imaging of Ministry of Education, School of Life Science and Technology, Xidian University, Xi’an, China; ^2^Assisted Reproduction Center, Northwest Women’s and Children’s Hospital, Xi’an, China; ^3^Department of Urology, Xijing Hospital, Fourth Military Medical University, Xi’an, China; ^4^Department of Andrology, Xiyuan Hospital, China Academy of Chinese Medical Sciences, Beijing, China

**Keywords:** lifelong premature ejaculation, feature selection, functional magnetic resonance imaging, support vector machine, functional connectivity

## Abstract

Recent neuroimaging studies have indicated that abnormalities in brain structure and function may play an important role in the etiology of lifelong premature ejaculation (LPE). LPE patients have exhibited aberrant cortical structure, altered brain network function and abnormal brain activation in response to erotic pictures. However, it remains unclear whether resting-state whole brain functional connectivity (FC) is altered in LPE patients. Machine learning analysis has the advantage of screening the best classification features from high-throughput data (such as FC), which has the potential to identify the pathophysiological targets of disease by establishing classification indicators for patients and healthy controls (HCs). Therefore, the supported vector machine based classification model using FC as features was used in the present study to confirm the most specific FCs that distinguish LPE patients from healthy controls. After feature selection, the remained features were used to build the classification model, with an accuracy 0.85 ± 0.14, sensitivity of 0.92 ± 0.18, specificity of 0.72 ± 0.30, and recall index of 0.85 ± 0.17 across 1000 testing groups (100 times 10-folds cross validation). After that, two-sample *t*-tests with family-wise error correction were used to compare these features that occur more than 500 times during training steps between LPE patients and HCs. Four FCs, (1) between left medial part of orbital frontal cortex (mOFC) and right mOFC, (2) between the left rectus and right postcentral gyrus, (3) between the right insula and left pallidum, and (4) between the right middle part of temporal pole and right inferior part of temporal gyrus showed significant group difference. These results demonstrate that resting-state brain FC might be a discriminating feature to distinguish LPE patients from HCs. These classification features, especially the FC between bilateral mOFC, provide underlying abnormal central functional targets in LPE etiology, which offers a novel alternative target for future intervention in LPE treatment.

## Introduction

In recent years, more and more neuroimaging studies have found that the etiology of sexual function dysfunction may be related to brain abnormalities, including brain structure, and functional aberrance (Zhao et al., 2015a, b; Chen et al., 2017; Jin et al., 2017; Li et al., 2018). Lifelong premature ejaculation (LPE) is one of the most common male sexual dysfunction diseases. According to the International Society for Sexual Medicine, LPE is defined as “a male sexual dysfunction characterized by ejaculation which always or nearly always occurs prior to or within about 1 min of vaginal penetration since the first sexual experience; and inability to delay ejaculation on all or nearly all vaginal penetrations; and negative personal consequences, such as distress, bother, frustration and or the avoidance of sexual intimacy (Althof et al., 2014).” Although selective serotonin reuptake inhibitors have been found to produce a side effect of delayed ejaculation in the treatment of depression and have gradually become the first-line drug for clinical treatment of premature ejaculation (PE) (Giuliano and Clement, 2012), the pathophysiological mechanisms of LPE remain unclear. As early as 10 years ago, neuroimaging studies demonstrated that the brain is involved in ejaculation behavior (Holstege et al., 2003; Georgiadis et al., 2007); however, evidence regarding to the role of the brain in the etiology of LPE remains limited, especially at the supraspinal level.

In Zhang et al. (2017), the first neuroimaging study of brain changes in LPE patients was conducted. Subsequently, there have been a few studies reporting brain structural and functional abnormalities in LPE, including by our group. These studies have shown that LPE patients have increased cortical thickness and possible improved sensory ascending conduction efficiency (Guo et al., 2017; Gao et al., 2018), and abnormal brain function either in resting state or during erotic picture stimulation (Zhang et al., 2017; Lu et al., 2018; Yang et al., 2018), which have provided new evidence for the neurobiological etiology of LPE. Recently, machine learning methods have also been used in the analysis of high-throughput brain imaging data to obtain more disease-specific imaging features. For example, classifiers based on brain structure or brain function features have been used to distinguish psychiatric patients from healthy people, to distinguish different subtypes of patients, and to predict remission and non-remission when evaluating therapeutic effects (Fu et al., 2008; Grotegerd et al., 2014; Redlich et al., 2016; Du et al., 2018). These classification features offer useful insight for detecting the biological mechanisms of diseases. Interestingly, a recent study investigating the brain mechanism of venous erectile dysfunction used machine learning classification to distinguish patients from healthy controls, and revealed more various white matter-derived indices that might underlie imaging targets related to the neurobiological etiology of venous erectile dysfunction (Li et al., 2018).

Therefore, in the present study, we aimed to use a machine learning method to classify LPE patients from healthy subjects based on high-throughput resting brain functional connectivity (FC) data, in effort to find the most specific discriminating indicators between LPE patients and healthy controls. We believe our results provide novel information for understanding the neurobiological mechanism of LPE.

## Materials and Methods

### Participants

Sixty male adults non-medicated PE patients and sixty male non-drug-using healthy controls (HCs) were recruited in our study. LPE was diagnosed according to the International Society for Sexual Medicine’s guidelines for the diagnosis and treatment of premature ejaculation (Althof et al., 2010). All participants underwent history taking and physical examination. Each patient had an intravaginal ejaculatory latency time (IELT) within 1 min. The premature ejaculation diagnostic tool (PEDT) score of each LPE patient was >11, but <5 for each control. The International Index of Erectile Function score was no less than 21 for all subjects. Participants were excluded if they met any of the following criteria: (1) had a history of alcohol or drug abuse, (2) had a history of psychiatric or neurologic diseases, (3) having a history of head trauma, and (4) had any contra-indication to MRI scanning.

According to the selection standards above, 45 PE patients and 40 HCs were included in the current study. Written informed consent was obtained from all study participants. Research procedures were approved by the ethical committee of the Northwest Women’s and Children’s Hospital in China, and were conducted in accordance with the Code of Ethics of the World Medical Association (Declaration of Helsinki).

### Imaging Data Acquisition

All subjects underwent a series of image scanning using a 3T GE MR750 scanner at the Department of Radiology, Xijing Hospital, the Fourth Military Medical University, Xi’an, China. A standard 8-channel head coil was used together with a restraining foam pad to minimize head motion and diminish scanner noise. Resting-state functional images were acquired with a single-shot gradient recalled echo planar imaging sequence. (TR/TE: 2000 ms/30 ms, field of view: 240 × 240 mm^2^, matrix size: 64 × 64, flip angle: 90°, in-plane resolution: 3.75 × 3.75 mm^2^, slice thickness: 3.5 mm with no gaps, 45 axial slices). For each subject, a total of 210 volumes were acquired. High resolution T1-weighted images were collected with a volumetric three-dimensional spoiled gradient recall sequence (TR/TE: 8.2 ms/3.18 ms, field of view: 256 × 256 mm^2^, matrix size: 512 × 512, flip angle = 9°, in-plane resolution: 0.5 × 0.5 mm^2^, slice thickness = 1 mm, 196 sagittal slices). During the resting scanning, subjects were instructed to keep their eyes open and to not think about anything.

### Imaging Data Preprocessing

Functional image preprocessing was carried out using CONN software^1^. Briefly, after excluding the first five images to ensure the signal had reached equilibrium, functional images were corrected for head motion and temporal differences. A participant was excluded if any translation or rotation parameters in subject’s data set exceeded ± 1 mm or ± 1°, respectively. After this step, 39 patients and 30 HCs remained. After that, outlier detection was performed. Next, the corrected functional images were coregistered to each subject’s T1 image without reslicing the image. After that, T1 images were normalized to the Montreal Neurological Institute (MNI) space, which generated a transformed matrix from native space to MNI space. Functional images were then transformed to the MNI space using this matrix and resampled at 2 × 2 × 2 mm^3^. Finally, all images were smoothed with a 6-mm full width at half maximum Gaussian kernel.

To remove spurious sources of variance, time series of each brain voxel were performed by the following steps: (1) linear detrending; (2) regressing out the six head motion parameters and their first-level derivative, the averaged cerebrospinal fluid and white matter signals, and the scrubbing signal from the time series generated by the functional outlier detection (ART-based identification of scans for scrubbing) process in CONN; (3) 0.01–0.1 Hz band-pass filtering.

After data preprocessing, time series of each region of interest (ROI) were extracted as the average time series across all voxels in that ROI based on the Anatomical Automatic Labeling (AAL) cortical and subcortical atlas (Tzourio-Mazoyer et al., 2002). In this step, 90 ROI time series were extracted. Finally, the FC coefficient (e.g., Pearson’s correlation coefficient) between each pair of these 90 time series was calculated, which resulted in 4005 edges for each subject for subsequent analysis.

### Features Selection and Classification Model

Ten-folds cross validation (CV) was used to assess the reliability of the classification model. Briefly, 69 subjects were randomly separated into 10 groups. Each time, one group in turn was used as a testing group and the other nine groups were used as training group.

Firstly, two sample *T*-test was used as the first step to preliminarily select features from the 4005 edges in training group. The edges with a *p*-value less than *P*_0_ were selected as initial features. After that, we used a 10-folds CV based Least Absolute Shrinkage and Selection Operator (CV-LASSO) method to further select features. Briefly, subjects in training group were again randomly separated into 10 groups. Each time, one group in turn was excluded from the dataset, and the LASSO (Sauerbrei et al., 2007) method with mean of square error (MSE) as the cost function was used on the remaining nine groups to narrow down the initial features into the most important features according to the MSE+1SE criteria (Sauerbrei et al., 2007). This step was repeated 10 times, which resulted in 10 different groups of selected features. Finally, the edges that were included in the selected feature group at least *N* times (i.e., occurring *N* times) were selected as LASSO features for further analysis. Next, the linear supported vector machine (LSVM) method was used to construct the classification model based on LASSO features in training group, which was implemented using *libsvm* software^2^. The accuracy, sensitivity, specificity and recall indices of the constructed model were calculated using testing group.

All these steps above were repeated 10 times. As for the setting of *P*_0_, *N*, and the cost parameter *c* in libsvm, we used grid-search method to find them. These parameters were set at a group of specific values when the accuracy index of the constructed classification model achieved the maximum. The *P*_0_ was set from 0.025 to 0.2 with a step of 0.025 and including 0.001, 0.005, and 0.01. The *N* was set from 1 to 10 with a step of 1. The *c* was set from 0.1 to 2 with a step of 0.1.

**FIGURE 1:**
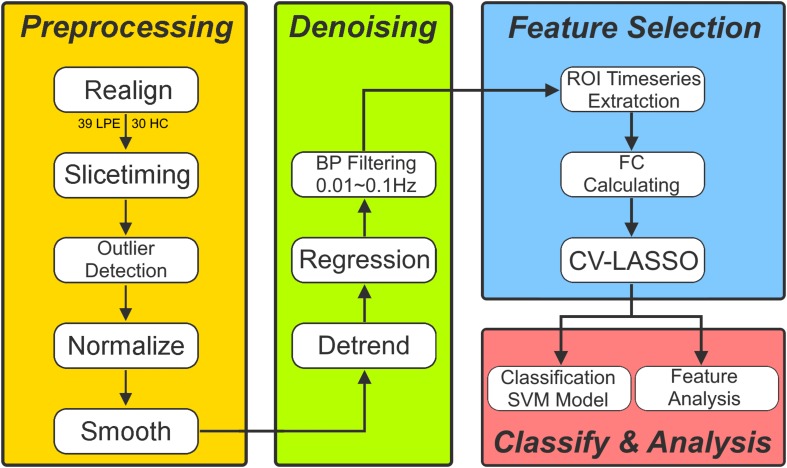
The framework of study procedure.

To avoid the random group effect, we repeated the 10-folds CV 100 times. For each time, a new random group was split. The mean ± standard deviation of each index across the 1000 testing groups (10 × 100) was used to assess the performance and stability of the constructed model. Finally, 1000 times permutation test (group label permutation) was performed to check if our results were significantly different from random label. Figure 1 illustrates the framework of our study.

## Results

### Clinical and Demographic Characteristics

Clinical and demographic characteristics of the subjects are shown in Table 1. The PEDT scores of LPE patients were significantly higher than those of HCs, and the IELT of LPE patients was dramatically shorter than that of HCs.

**TABLE 1:** Clinical and demographic characteristics.

	**HC (*n* = 30)**	**LPE (*n* = 39)**	***P*-value**
Age (years)	31.33 ± 2.77	30.52 ± 5.06	0.44
PEDT score	0.80 ± 1.40	17.50 ± 1.96	<0.0001
IIEF-5 score	24.5 ± 0.63	24.29 ± 0.47	0.17
IELT (min)	644.00 ± 366.47	37.02 ± 16.75	<0.0001

*Data were presented as mean ± SD. HC, healthy control; IELT, intravaginal ejaculatory latency time; IIEF-5, International Index of Erectile Function-5; LPE, lifelong premature ejaculation; PEDT, Premature ejaculation diagnostic tool.*

### Classification

**FIGURE 2:**
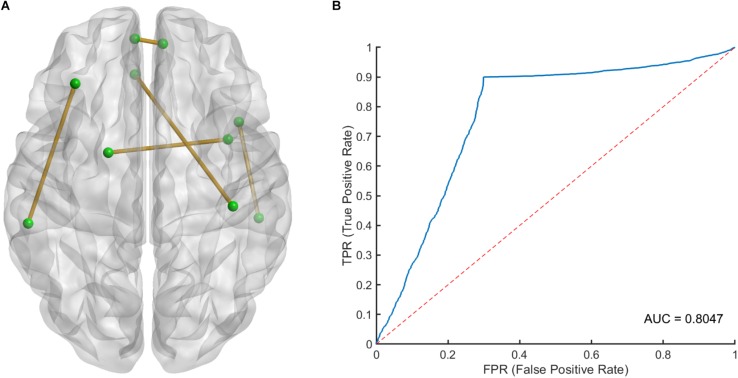
**(A)** The spatial distribution of five selected LASSO features and **(B)** the receiver operating characteristic (ROC) curve of the classification model. LASSO, least absolute shrinkage and selection operator.

**FIGURE 3:**
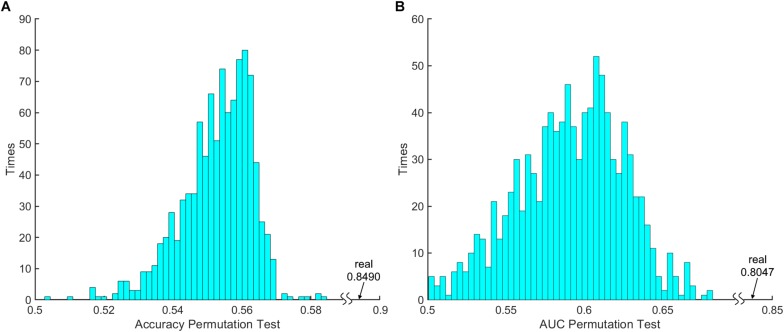
The 1000 times permutation test results of **(A)** classification model accuracy index and **(B)** area under curve (AUC).

The 100 times 10-fold CV results of the model were shown in Table 2. The accuracy, sensitivity, specificity and recall indices of the classification model were 0.8490 ± 0.1401, 0.9238 ± 0.1817, 0.7250 ± 0.3038, and 0.8506 ± 0.1740, respectively. Figure 2B displays the receiver operating characteristic curve (ROC) of the classification model, and the AUC was 0.8047. Figure 3 shows the permutation test results of our constructed classification model. Together, these results demonstrate the stability of our classification model and the reliability of our method.

**TABLE 2:** Performance information of classification model.

	**Accuracy**	**Sensitivity**	**Specificity**	**Recall**	**AUC**
	0.8490 ± 0.1401	0.9238 ± 0.1817	0.7250 ± 0.3038	0.8506 ± 0.1740	0.8047
Permutation	< 0.001	–	–	–	<0.001

*AUC, area under the curve; Permutation, 1000 Permutation test.*

After counting the occurring time of each LASSO feature in 100 times 10-fold CV, 5 LASSO features with occurring time larger than 500 were finally selected as the most important features in classification, which involved brain regions in the frontal, temporal and limbic lobes (Table 3), since we believed that features which occurring time less than 500 were to a large extent relied on the specific splitting group. Figure 2A gives the spatial distribution of these 5 LASSO features.

**TABLE 3:** Detailed information of five selected LASSO features.

**Edge**		**Weight**
**HC>PE**		
Frontal_Med_Orb_L	Frontal_Med_Orb_R	0.4874
Rectus_L	Postcentral_R	0.0020
Insula_R	Pallidum_L	0.1270
**HC<PE**		
Frontal_Mid_L	SupraMarginal_L	0.1370
Temporal_Pole_Mid_R	Temporal_Inf_R	0.2466

*Frontal_Med_Orb, medial part of orbital frontal cortex; Frontal_Mid, middle part of frontal cortex; Temporal_Pole_Mid, middle part of temporal pole; Temporal_Inf, inferior part of temporal gyrus; L for left and R for right; LASSO, absolute shrinkage and selection operator.*

## Discussion

By using a machine learning classification method to assess resting-state brain function in LPE patients, the present study screened 9.042 (average across every training step during 100 times 10-folds CV) out of 4005 FC features to construct the optimal classifier, which could separate patients from healthy people with an accuracy of 0.85. These FC features are mainly distributed in some areas in the frontal, temporal, and parietal cortex, and limbic system. Compared with previous studies, our results provide more novel FC-derived indicators through a strategy of classification research to understand the potential abnormalities of brain function in LPE patients.

The classification algorithm in machine learning is useful for exploring the best classification features from high-throughput information, in which multivariate decoding algorithms like supported vector machine are trained on a portion of the data by weighting all connections in order to separate the known clinical status from HCs, rather than testing each connection independently for group differences. The whole brain functional connections belong to a high-throughput data set, in which there are more than 4000 FCs in the whole brain when the human brain is divided into 90 ROIs. In our present study, through CV-LASSO dimension reduction method, we have obtained a classifier with a relatively high accuracy to individually distinguish LPE patients from HCs. This machine learning-based classification approach based on resting-state FC has previously been used to distinguish patients with brain disorders from HCs, and responders from non-responders in clinical drug or invention trials (van Waarde et al., 2015; Sarpal et al., 2016; Arbabshirani et al., 2017; Plaschke et al., 2017). Therefore, the features based on the resting-state FC in our present study may be biomarkers that allow the classification of individual LPE patients.

Among the five selected features which occur more than 500 times in training step during 100 times 10-fold CV, the connections between bilateral mOFC had the highest weight according to our results. OFC has been implicated in ejaculation control. A previous positron emission tomography study has reported a remarkable decrease of regional cerebral blood flow throughout the prefrontal cortex during ejaculation in male volunteers (Holstege et al., 2003; Georgiadis et al., 2007). Our previous study has also found abnormal prefrontal control function in LPE patients by using classical inhibitory control tasks, and reduced FC between the inferior frontal cortex and the frontal pole was found in LPE patients (Yang et al., 2018). Together with the present results that the synchronized activity of the mirror symmetric OFC had absolute superiority in discriminating LPE patients from the healthy controls, it further indicates that the OFC is likely closely involved in the etiology of LPE, and the OFC-related inhibitory control function may be impaired in LPE patients, which might cause the loss of the inhibitory tone on ejaculation in LPE patients.

Besides, most of the other FC related regions in the current study were also reported to be related to male sexual physiology. Zhang et al. (2017) have found that the insula and middle part of temporal gyrus showed abnormal activation in response to erotic stimulation, and also had aberrant regional activity

and FC during resting state in LPE patients. By using cerebral cortical thickness measurements, we once reported widespread cortical thickening in the orbitofrontal, middle frontal, and supramarginal gyrus in LPE patients (Guo et al., 2017). A recent fMRI study detected the resting-state FC density in LPE patients, which found that anterior cingulate cortex, insula, and precuneus had increased long-range FC density in LPE patients compared to healthy controls (Lu et al., 2018). Although the role of gyrus rectus and postcentral gyrus in LPE has not been reported yet, the gyrus rectus is located in the medial orbital gyrus and plays an inhibition role in sexual arousal (Stoleru et al., 2012), and sensory stimuli from penis could induce Rolandic opercula area and postcentral gyrus activation (Stoleru et al., 2012).

So, despite our results were derived from a data-driven method, these classification features that are involved in ejaculation and other sexual behaviors extend our knowledge of the central pathophysiology in LPE patients.

There are several limitations in the current study. We only included LPE patients without secondary PE patients. So, we do not know if our classifier was specific to LPE or trans-disease subtypes across all PE patients. Further research is necessary to include more subtypes of PE patients for classification studies. In addition, other than FC, brain gray matter and white matter structure have often been used as classification indicators. Li et al. (2018) have used white matter indicators to successfully distinguish venous erectile dysfunction patients from HCs. These measures were not included in the present study, but multimodal brain imaging information should be used in future classification studies of PE.

## Conclusion

By using machine learning analysis, this study identified potential neuroimaging markers based on resting-state whole brain FC that could distinguish LPE patients from HCs. These classification features provide novel information for explaining the central mechanisms of LPE, and further emphasize the potential functional abnormalities of the central inhibitory control network and sexual-related regions in LPE patients.

## Ethics Statement

Written informed consent was obtained from all study participants. Research procedures were approved by the ethical committee of the Northwest Women’s and Children’s Hospital in China, and were conducted in accordance with the Code of Ethics of the World Medical Association (Declaration of Helsinki).

## Author Contributions

JS, PL, XY, and WQ contributed to the conception and design. LL contributed to the acquisition of data. ZX and XY contributed to the data analysis and manuscript writing. XY, LL, and ZX contributed to the interpretation of the results.

## Conflict of Interest Statement

The authors declare that the research was conducted in the absence of any commercial or financial relationships that could be construed as a potential conflict of interest.

## Footnotes

1 http://web.mit.edu/swg/software.htm

2 https://www.csie.ntu.edu.tw/~cjlin/libsvm/
